# Diagnostic accuracy of magnetic resonance imaging in detection of intra-axial gliomas

**DOI:** 10.12669/pjms.37.1.2489

**Published:** 2021

**Authors:** Sohbia Munir, Sohail Ahmed Khan, Hina Hanif, Maria Khan

**Affiliations:** 1 Sohbia Munir, Resident, Dow Institute of Radiology, Dow University of Health Sciences, Karachi, Pakistan; 2 Sohail Ahmed Khan, Assistant Professor, Dow Institute of Radiology, Dow University of Health Sciences, Karachi, Pakistan; 3 Hina Hanif, Resident, Dow Institute of Radiology, Dow University of Health Sciences, Karachi, Pakistan; 4 Maria Khan, Resident, Dow Institute of Radiology, Dow University of Health Sciences, Karachi, Pakistan

**Keywords:** Diagnostic accuracy, Glioma, Magnetic resonance imaging, Specificity, Sensitivity

## Abstract

**Objective:**

To evaluate the diagnostic accuracy of magnetic resonance imaging (MRI) in detection of intra-axial gliomas in suspected cases keeping histopathology as gold standard.

**Methods:**

This cross-sectional study was conducted at Dow Institute of Radiology, DUHS from October 2017 - April 2018. Patients of either gender aged 30-70 years presenting with headache were included. Patients already diagnosed and referred for follow up were excluded. MRI was performed on 1.5T scanner by a trained MRI technician. T1, T2, FLAIR, diffusion weighted and T1 post contrast images were acquired and reviewed by two radiologists having more than five years post fellowship experience. Sensitivity, specificity, PPV, NPV and diagnostic accuracy of MRI for intraaxial gliomas was calculated taking histopathology findings as gold standard.

**Results:**

Mean age of the patient`s was 51.71 ±10.85 years. Positive intraaxial gliomas on MRI were observed in 123 (79.90%) patients while on histopathology, positive intraaxial gliomas were observed in 131 (85.10%) patients. Diagnostic accuracy of MRI in detection of intra-axial gliomas taking histopathology findings as gold standard showed sensitivity, specificity, positive predicted value (PPV), negative predicted value (NPV) and overall diagnostic accuracy as 89.31%, 73.91%, 95.12%, 54.84% and 87.01%.

**Conclusions:**

MRI has high sensitivity, moderate specificity and high diagnostic accuracy in detection of intraaxial gliomas.

## INTRODUCTION

Gliomas are the most common intrinsic tumours of the central nervous system and are graded according to the World Health Organization (WHO), from Grade-I to Grade-IV, with increasing malignancy.^1^-^3^ The incidence of brain tumors has increased in recent several years, and the incidence shows significant difference in terms of gender, age, race, ethnicity, and even geographical region.^4^

Paediatric brainstem gliomas (BSG) account for 10-20% of CNS neoplasms in children.^5^ They are characterized by infiltrative growth of tumor cells, including along white matter tracts.^6^,^7^ Contrast enhanced MRI is the mainstay for imaging cerebral tumors.^8^,^9^ The general prognosis for patients is very poor, particularly for the elderly patients. The mechanisms of carcinogenesis for glioma are still not fully understood. Evidence suggests that exposure to radiation might be an important risk factor for glioma, which could explain a small proportion of glioma because the exposure is generally rare. Accurate grading of tumors is critical for planning therapeutic strategies, assessing prognosis, and monitoring response to therapy.^10^ Sampling errors and improper grading pose a difficulty due to heteroegeneity of the tumors due to internal necrotic components. An ample amount of data is already present regarding the accuracy of MRI for brain gliomas however most of the previous local studies are carried out on a smaller sample size.^11^ Our objective was to evaluate the diagnostic accuracy of magnetic resonance imaging (MRI) in detection of intra-axial gliomas in suspected cases keeping histopathology as gold standard.

## METHODS

A cross sectional study was conducted at Dow Institute of Radiology (DIR), Dow University of Health Sciences, Ojha campus, Karachi from October 2017 - April 2018. This dissertation-based article was approved from the Research Evaluation Unit of College of Physicians and Surgeons of Pakistan was obtained prior conducting of study (Ref #: CPSP/REU/RAD-2015-256-2040).

All patient aged 30-70 years of either gender suspected of having intra-axial gliomas presenting with headache and fits for more than 15 days were enrolled. While those patients already diagnosed as having intra-axial gliomas and came for follow-up, pregnant women, patients allergic to intravenous contrast and abnormal renal function tests and all patients who were claustrophobic to MRI were excluded.

This study was conducted in large tertiary care public sector in radiology institution.Sample size was calculated using prevalence of intra-axial glioma as 69%^11^, sensitivity of MRI: 93%^11^, specificity of MRI: 77%^11^ with margin of error 5% for sensitivity and 10% for specificity, Confidence level of 95%. The final sample size came to be of 154 patients.

On MRI, intra-axial gliomas were labeled as the presence any of the following features: poorly circumscribed lesion in brain parenchyma showing marked perilesional edema, hypo to isointense on T1W images, hyperintense on T2W images and FLAIR, showing diffusion restriction on DW images and showing contrast enhancement.

On histopathology, presence of any three of the following on histopathology were marked as intra-axial gliomas positive: high cellularity, cellular and nuclear anaplasia, necrosis and microvascular proliferation, proportion of mitotic figure seen on microscopy

MRI scan of brain of the selected patients was performed on SIGNA EXPLORER GE 1.5 TESLA MRI. Axial, sagittal and coronal images of T1 weighted, T2 weighted, Diffusion weighted, FLAIR sequences and post gadolinium T1 weighted were obtained in all the patients and reported by senior radiologists having more than five years’ post fellowship experience. Later histopathology was followed after surgery.

Presence of intra-axial gliomas detected by both MRI and histopathology was labeled as true positive. Presence of intra-axial gliomas detected only by MRI and not by histopathology was labeled as false positive. No intra-axial gliomas detected by both MRI and histopathology was labeled as true negative. Presence of intra-axial gliomas detected by histopathology and not by MRI was labeled as false negative.

Data was analyzed by using SPSS version 22. Mean and standard deviations were computed for quantitative variables like age and duration of symptoms. Frequency and percentages were calculated for gender, findings on MRI and histopathology. Diagnostic accuracy was evaluated by calculating sensitivity, specificity, positive and negative predictive values.

## RESULTS

**Fig.1:**
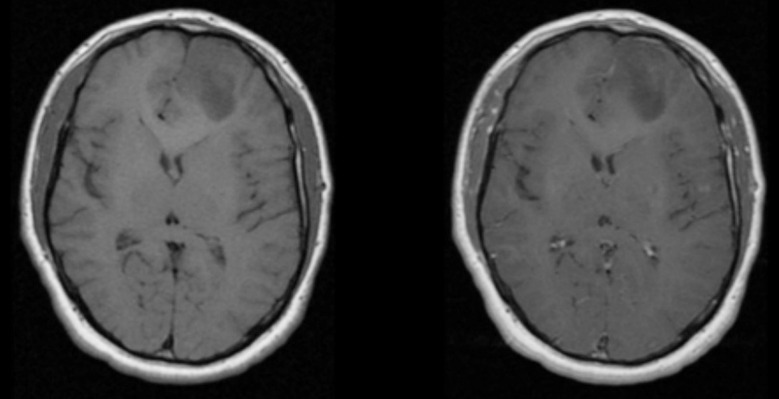
T1 and T1 post contrast of Diffuse Astrocytoma. Abnormal signal intensity area noted in left frontal lobe appearing as hypointense on T1WI and shows no significant enhancement on contrast study

**Fig.2:**
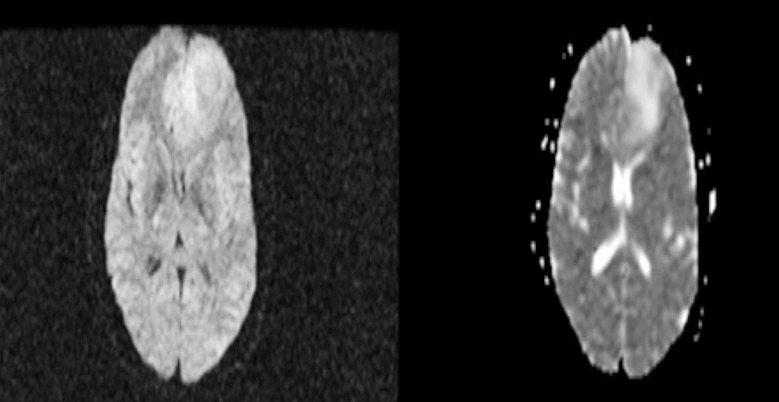
DWI and ADC of Diffuse Astrocytoma. The tumor in left frontal lobe show no diffusion restriction.

Of 154 patients, mean age was 51.71 ± 10.85 years. There were 72 (46.80%) patients with ≤50 years of age and 82 (53.20%) with >50 years of age. Majority of the patients were males (n=106, 68.80%) and 48 (31.20%) were females. Mean duration of symptoms was 36.81 ± 15.46 days. There were 82 (53.20) patients with ≤35 days of duration of symptoms and 72 (46.80%) patients with >35 days of duration of symptoms. Positive intraaxial gliomas on MRI were observed in 123 (79.90%) patients while on histopathology positive intraaxial gliomas were observed in 131 (85.10%) patients. (Fig. 1 and 2)

Diagnostic accuracy of MRI in detection of intra-axial gliomas taking histopathology findings as gold standard showed sensitivity, specificity, positive predicted value (PPV), negative predicted value (NPV) and overall diagnostic accuracy as 89.31%, 73.91%, 95.12%, 54.84% and 87.01% respectively (Table-I).

**Table-I:** Diagnostic accuracy of MRI taking Histopathology as gold standard (n=154).

*MRI*	*Histopathology*		

	Positive	Negative	Total
Positive	117	6	123
Negative	14	17	31
Total	131	23	154

Sensitivity: 89.31%, Specificity: 73.91%, PPV: 95.12%, NPV: 54.84%, Overall diagnostic accuracy: 87.01%.

## DISCUSSION

Gliomas are among the commonest tumors involving the brain.^12^ Evaluation of gliomas is generally undertaken by utilization of MRI. The current standard mode of treatment for gliomas include surgical resection and chemotherapeutic agents along with radiation therapy.^13^ Imaging is utilized to monitor the treatment effectiveness.^14^ Decisions about continuation / discontinuation of a treatment regimen rests upon effective imaging. The tumor is generally imaged by MRI with administration of gadolinium. T1-weighted, T2-weighted and T1-weighted post gadolinium enhancement sequences are important for evaluation of detection of glioma.

**Fig.3:**
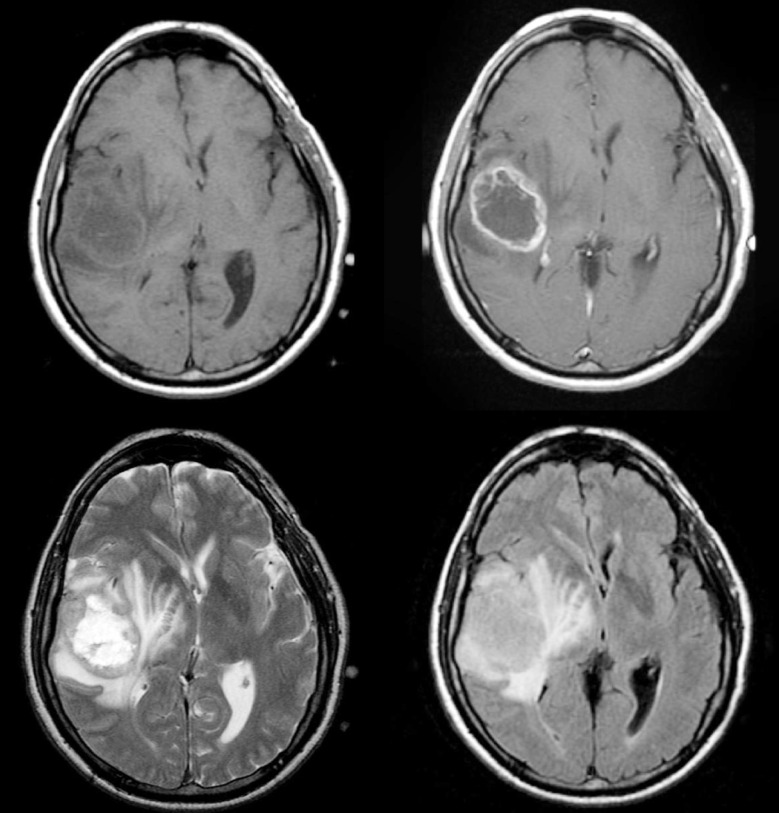
MRI of glioblastoma. A large mass in the right parietotemporal lobe causing significant mass effect with adjacent vasogenic oedema. It appears heterogeneously low on T1, high on T2 and irregular peripheral nodular enhancement. It has a central non-enhancing necrotic component.

**Table-II:** Baseline characteristics and diagnostic accuracy of MRI taking histopathology as gold standard (n=154).

	N	Sensitivity	Specificity	PPV	NPV	Overall Diagnostic Accuracy

***Age, years***						
≤50	72	88.14%	76.92%	94.55%	58.82%	86.11%
>50	82	90.28%	70.00%	95.59%	50.00%	87.80%
***Gender***						
Male	106	87.78%	81.25%	96.34%	54.17%	86.79%
Female	48	92.68%	57.14%	92.68%	57.14%	87.50%
***Duration of symptoms, days***						
≤35	82	87.32%	90.91%	98.41%	52.63%	87.80%
>35	72	91.67%	58.33%	91.67%	58.33%	86.11%

In Pakistan, the gliomas are not uncommon.^11^ Some of their common clinical manifestation include headache, vomiting, and seizures. Change in personality, vision disturbance, feeling of vertigo or dizziness and hemiparesis may also be the presenting features.^15^ MR imaging provides important information related to enhancement by administration of gadolinium contrast material, perilesional edema, foci of tumor having distant spread, hemorrhage within / surrounding the tumor, necrosis within the tumor, mass effect caused by the tumor and the resultant midline shift. These features are helpful in characterizing tumor aggressiveness and hence tumor grade.^16^

Clinical management and assessment of prognosis is dependent upon the grade of gliomas. Assessment of glioma grade before surgery is also important for the therapeutic decision making. Low grade gliomas are the Grade-I tumors that have slow progression. They are usually considered benign. Grade-II gliomas have atypial at the nuclear level. These tumors have a low cellular count as well as reduced amount of vascularity. Grade-II tumor cells are sometimes mixed with normal brain parenchyma. On MRI the low-grade gliomas appear as a homogeneous lesion. Contrast enhancement is uncommon. Perilesional edema may be present. Grade-III gliomas are anaplastic and Grade-IV gliomas have high cellularity. Their vascularity is also higher and they have an increased amount of necrotic component. Heterogeneous enhancement is usually appreciated on contrast administration. However, imaging features may overlap in findings.^17^

Sensitivity, Specificity, PPV and NPV of MRI in detecting intra-axial gliomas were 93%, 77%, 80% and 90% by taking histopathology as gold standard while prevalence of intra-axial glioma was 69% as documented by Chishty et al. in 2010.^11^

The results of present study have shown that MRI has a high sensitivity, moderate specificity and high diagnostic accuracy in diagnosis of intra axial gliomas. Current study has slightly lower sensitivity than the one reported in the previous study.^11^ This can be secondary to variation in cellularity of the tumor. Moreover, dose of contrast used and the time of scanning potentially may have an effect on the sensitivity.

Study showed that MRI has moderate specificity for diagnosis of intraaxial gliomas. The specificity reported in our study is slightly higher as reported in another study.^11^ The reason for this high specificity could be due to better depiction of solid enhancing and cystic component and other tumor feature for exact evaluation of glioma.

Recent advances in MRI have shown the way for development of new advanced imaging sequences for evaluation of intraaxial glioma. Magnetic resonance spectroscopy (MRS) is developing as a new modality for glioma diagnosis.^18^,^19^ Diffusion Weighted Imaging (DWI) along with apparent diffusion coefficient (ADC) mapping also has a role in evaluation of brain gliomas.^20^ Susceptibility weighted imaging (SWI) and perfusion MRI also have a promising role.^21^,^22^

### Limitations of the study

Firstly, the study duration was short. Moreover, inter observer and intraobserver variability was not evaluated. Despite these limitations, a strength of our study was prospective collection of data from the participants involved in this study.

## CONCLUSION

Gliomas are among the commonest tumors involving the brain. Evaluation of gliomas is generally undertaken by utilization of contrast enhanced MRI which has a high sensitivity, moderate specificity and a high diagnostic accuracy in detection of intra-axial gliomas.

### Recommendation

It is recommended that further multi-centeric studies should be carried out in the future on a larger sample size to provide an accurate depiction of utilization of MRI for intraaxial gliomas. Moreover, interobserver and intraobserver variability should be calculated so as to evaluate the considerable variability and overcome the reporting deficiencies. Furthermore, variables such as quality of life and performance status of the patients shall be included in future studies to evaluate the patient response to the treatment regimens.

### Authors’ Contribution:

**SM** conceived, designed and did statistical analysis & editing of manuscript. **HF, MK, SAK** did data collection and manuscript writing. **SM, SAK** takes the responsibility and is accountable for all aspects of the work in ensuring that questions related to the accuracy or integrity of any part of the work are appropriately investigated and resolved.
